# Supplementary material for: Comparative genomics provides new insights into the diversity, physiology, and sexuality of the only industrially exploited tremellomycete: Phaffia rhodozyma
Source: BMC Genomics. 2016 Nov 9;17:901. doi: 10.1186/s12864-016-3244-7 (PMC5103461; doi:10.1186/s12864-016-3244-7)
Supplement: Additional file 6: — List of orphan genes with links to PFAM (related to Additional file 1: Table S1). (ZIP 1428 kb) [file 12864_2016_3244_MOESM6_ESM.zip › PFAM_UNIQUE/G02866_P.html]

#                                                                            --- full sequence --- -------------- this domain -------------   hmm coord   ali coord   env coord
  
# target name        accession   tlen query name           accession   qlen   E-value  score  bias   #  of  c-Evalue  i-Evalue  score  bias  from    to  from    to  from    to  acc description of target
  
#------------------- ---------- ----- -------------------- ---------- ----- --------- ------ ----- --- --- --------- --------- ------ ----- ----- ----- ----- ----- ----- ----- ---- ---------------------
  
G02866\_P             -            438 BatD                 PF13584.1    485      0.06   10.6   0.1   1   1   1.6e-05     0.098    9.9   0.1   380   455   128   202   110   209 0.69 - Oxygen tolerance  
G02866\_P             -            438 DAP10                PF07213.6     79     0.068   11.6   0.2   1   1   3.1e-05      0.19   10.2   0.2    18    61   154   199   148   214 0.71 - DAP10 membrane protein  
G02866\_P             -            438 DDHD                 PF02862.12   227     0.091   11.2   0.6   1   1   0.00047      0.19   10.2   0.6    67   150    74   156    68   239 0.66 - DDHD domain  
G02866\_P             -            438 DUF1517              PF07466.6    289       0.1   10.2   1.3   1   1   0.00015       0.3    8.7   1.3    17    94   130   202   114   223 0.52 - Protein of unknown function (DUF1517)  
G02866\_P             -            438 DUF3595              PF12166.3    422      0.72    7.3   5.2   1   1   0.00057       1.1    6.6   5.2   216   358    44   191    11   202 0.57 - Protein of unknown function (DUF3595)  
G02866\_P             -            438 DUF912               PF06024.7    101     0.031   13.0   0.0   1   1   6.8e-05     0.068   11.9   0.0    29    93   137   199   106   208 0.53 - Nucleopolyhedrovirus protein of unknown function (DUF912)  
G02866\_P             -            438 Herpes\_gE            PF02480.11   439      0.04   10.8   4.0   1   2     0.097        53    0.6   1.5   327   404    10    89     2   140 0.75 - Alphaherpesvirus glycoprotein E  
G02866\_P             -            438 Herpes\_gE            PF02480.11   439      0.04   10.8   4.0   2   2   7.7e-05     0.042   10.8   0.1   329   396   144   210   114   240 0.73 - Alphaherpesvirus glycoprotein E  
G02866\_P             -            438 Kei1                 PF08552.6    189       2.9    6.2   5.7   1   2      0.26   7.8e+02   -1.7   0.3   125   141    84   100    35   125 0.44 - Inositolphosphorylceramide synthase subunit Kei1  
G02866\_P             -            438 Kei1                 PF08552.6    189       2.9    6.2   5.7   2   2   0.00045       1.3    7.3   3.1   108   184   127   202    95   206 0.62 - Inositolphosphorylceramide synthase subunit Kei1  
G02866\_P             -            438 Macoilin             PF09726.4    697       9.9    2.9  16.2   1   1     0.097        13    2.6  16.2   282   378    70   163     2   178 0.62 - Transmembrane protein  
G02866\_P             -            438 MFS\_1                PF07690.11   352      0.11    9.8   0.0   1   2       6.2     3e+02   -1.4   2.1   280   322    36    76    12   105 0.43 - Major Facilitator Superfamily  
G02866\_P             -            438 MFS\_1                PF07690.11   352      0.11    9.8   0.0   2   2    0.0023      0.11    9.8   0.0   102   188   121   207   115   259 0.78 - Major Facilitator Superfamily  
G02866\_P             -            438 Peptidase\_S64        PF08192.6    695       9.9    3.0  11.0   1   1     0.035        14    2.5  11.0    30   135    61   166    40   181 0.67 - Peptidase family S64  
G02866\_P             -            438 RCR                  PF12273.3    130      0.55    9.5  10.4   1   2    0.0013       1.3    8.2   0.0     3    31   175   204   175   299 0.83 - Chitin synthesis regulation, resistance to Congo red  
G02866\_P             -            438 RCR                  PF12273.3    130      0.55    9.5  10.4   2   2       2.4   2.4e+03   -2.3  11.2    46   107   359   425   332   436 0.49 - Chitin synthesis regulation, resistance to Congo red  
G02866\_P             -            438 Rifin\_STEVOR         PF02009.11   299      0.41    8.9   9.1   1   1    0.0019      0.89    7.8   9.0   139   283    54   197    17   206 0.54 - Rifin/stevor family  
G02866\_P             -            438 TrbL                 PF04610.9    225     0.021   13.2   6.4   1   1   1.5e-05     0.011   14.1   4.5    17   120    86   192    70   195 0.67 - TrbL/VirB6 plasmid conjugal transfer protein
